# A practical approach of salt and protein restriction for CKD patients in Japan

**DOI:** 10.1186/s12882-016-0298-3

**Published:** 2016-07-19

**Authors:** Kunitoshi Iseki, Kunihiro Yamagata

**Affiliations:** Clinical Research Support Center, Tomishiro Central Hospital, Tomigusuku, Okinawa Japan; Department of Nephrology, Faculty of Medicine, University of Tsukuba, Tsukuba, Ibaraki Japan; Division of Nephrology, 1-1-1 Tennodai, Tsukuba, Ibaraki 305-8577 Japan

## Abstract

Dietary management, in particular salt and protein restriction is fundamental for the proper care of CKD patients. Therefore, a practical guide to the dietary treatment may be helpful among progressive CKD patients. In Japan, two academic societies such as Japanese Society of Nephrology and Japanese Society for Dialysis Therapy have recently published Guidelines for Dietary Management for non-dialysis dependent CKD and dialysis-dependent CKD, respectively.

In this manuscript, we summarized the practical guide for salt and protein restriction from the Japanese Society of Nephrology to retard the progression of CKD to endstage renal disease. This guide will promote further the collaboration of Nephrologists and Dietitians.

## Background

CKD is common and the number of patients on renal replacement therapy (RRT) is estimated more than 330,000 in Japan [1]. It is becoming a social and economic burden. More than half of incident dialysis patients are CKD associated with diabetes mellitus (DM) and hypertension. Early detection and treatment would be necessary to retard the progression of CKD. Therefore, life-style modification including dietary management is important in patients with DM, hypertension, dyslipidemia, obesity, metabolic syndrome, and hyperuricemia. Among the dietary management, salt and protein restriction are critical for CKD patients, however it is influenced by differences in ethnic, regional, and cultural factors. We recently summarized a manual for dietician and other medical staff concerning life-style and dietary management in CKD patients (written in Japanese) [2, 3].

## Discussion

### Manual for dietary management

Table 1 summarized the guidance for lifestyle and dietary modification in CKD patients. Firstly, it is important diagnose the current stage of CKD (eGFR and proteinuria), cause of CKD, and also it is helpful to obtain the recent trend in CKD progression, pattern and slope of eGFR decline. Check the current medication and adherence to the drug therapy. Cessation of smoking and weight control should be accompanied with salt restriction and protein restriction. Until favorable results obtained, several rounds will be needed (Table 2).

**Table 1:** Guidance for Lifestyle and dietary modification in CKD patients (Cited with permission from the Japanese Society of Nephrology)

1. Lifestyle and Dietary Modification	
2. Weight control: BMI < 25.0 kg/m^2^	
3. Salt restriction: NaCl 3 to 6 g/day (if hypertensive)	Target blood pressure: <130/80 mmHg
4. Protein restriction (Body weight as body mass index 22.0 kg/m^2^)	Stage 3a: 0.8 to 1.0 g/kg/day
	Stage 3b: 0.6 to 0.8 g/kg/day
	Stage 4/5: 0.6 to 0.8 g/kg/day
	Stage 5D: HD patient 0.9 to 1.2 g/kg/day
	PD patient 0.9 to 1.2 g/kg/day
5. Potassium restriction (if hyperkalemia)	Stage 3b: ≤2000 mg/day
	Stage 4/5: ≤1500 mg/day
6. Glucose (if DM present): HbA1c < 7.0 %	
7. Lipids (if dyslipidemia): LDL-C < 120 mg/dL

**Table 2:** Flow chart for Lifestyle and dietary modification (Cited with permission from the Japanese Society of Nephrology)

1^st^ Round	1) Obtain good rapport with patient
	2) Make a checklist for individual patient
	3) Clear the priority (What is the category in the checklist?)
For: Protein restriction, Salt restriction	Use the manual of Guidance (30 min in each guidance)
For: Weight control, Hypertension, Hyperglycemia	Hyperkalemia, Smoking cessation, Hyperlipidemia, Hyperuricemia, Use algorithm (30 min in each guidance)
2^nd^ Round: If not satisfactory, repeat the guidance again	If the first priority was successful, then try second
	3rd Round: If not satisfactory, repeat the guidance again (Continue counselling)
	If the second priority was not-successful, then repeat the guidance again

### Steps for salt and protein restriction

1. Use the checklist
  Obtain clinical and laboratory information of the patient.
2. Check the current problem
  Adherence to drug therapy should be checked. Important categories are body mass index (BMI), blood pressure, fasting blood glucose, LDL-cholesterol.
3. Guidance by category
  Salt (NaCl) Restriction, Protein Restriction
4. Guidance by algorithm
  BMI, Blood Pressure, Blood Glucose, Lipid, Smoking Cessation, Potassium, Uric Acid
5. Useful materials
  Salt content in food staff

### Salt restriction

**Fig. 1:**
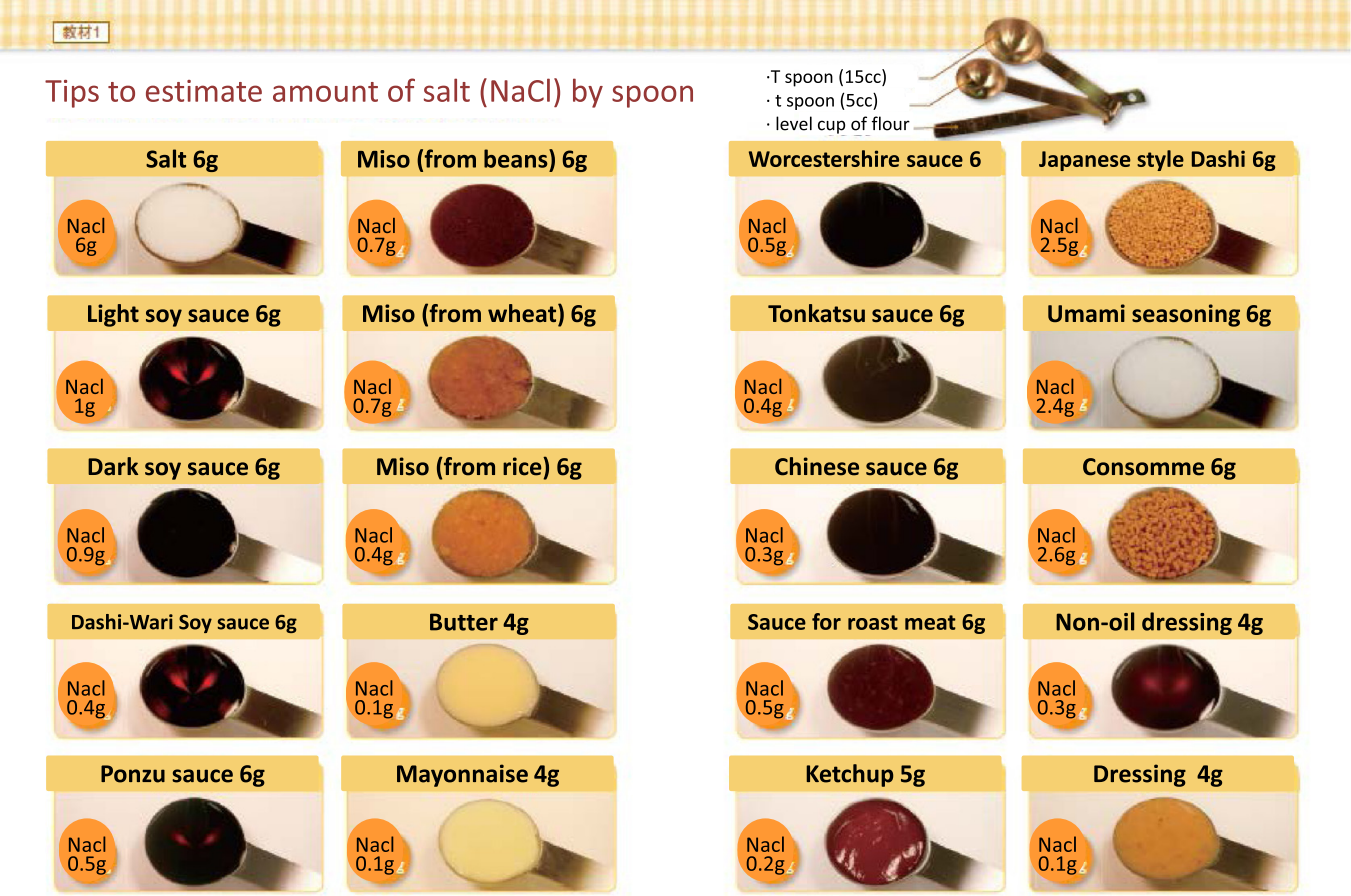
Estimating of salt content in the seasoning material
  Use spoon to estimate the amount of added seasoning, in particular when uses common seasoning materials such as table salt, source, and Miso.
  Estimate roughly as salt (gram) per one small spoon.
  Select low salt seasoning and law salt food staffs (Avoid too much).
  Check the salt ingredient in each food staff.
- Salt content in processed food (Fig. 2)

1. Rationale
  Salt restriction is essential for CKD patients. If not adequately controlled, salt retention may cause edema, heart failure and hypertension. Daily intake from foods and additives should be estimated carefully. In particular, salt intake may vary with cooking process.
2. Practical Guide
  We summarized several tools to help adhering to salt restriction.

- Salt content in seasoning (Fig. 1)

**Fig. 2:**
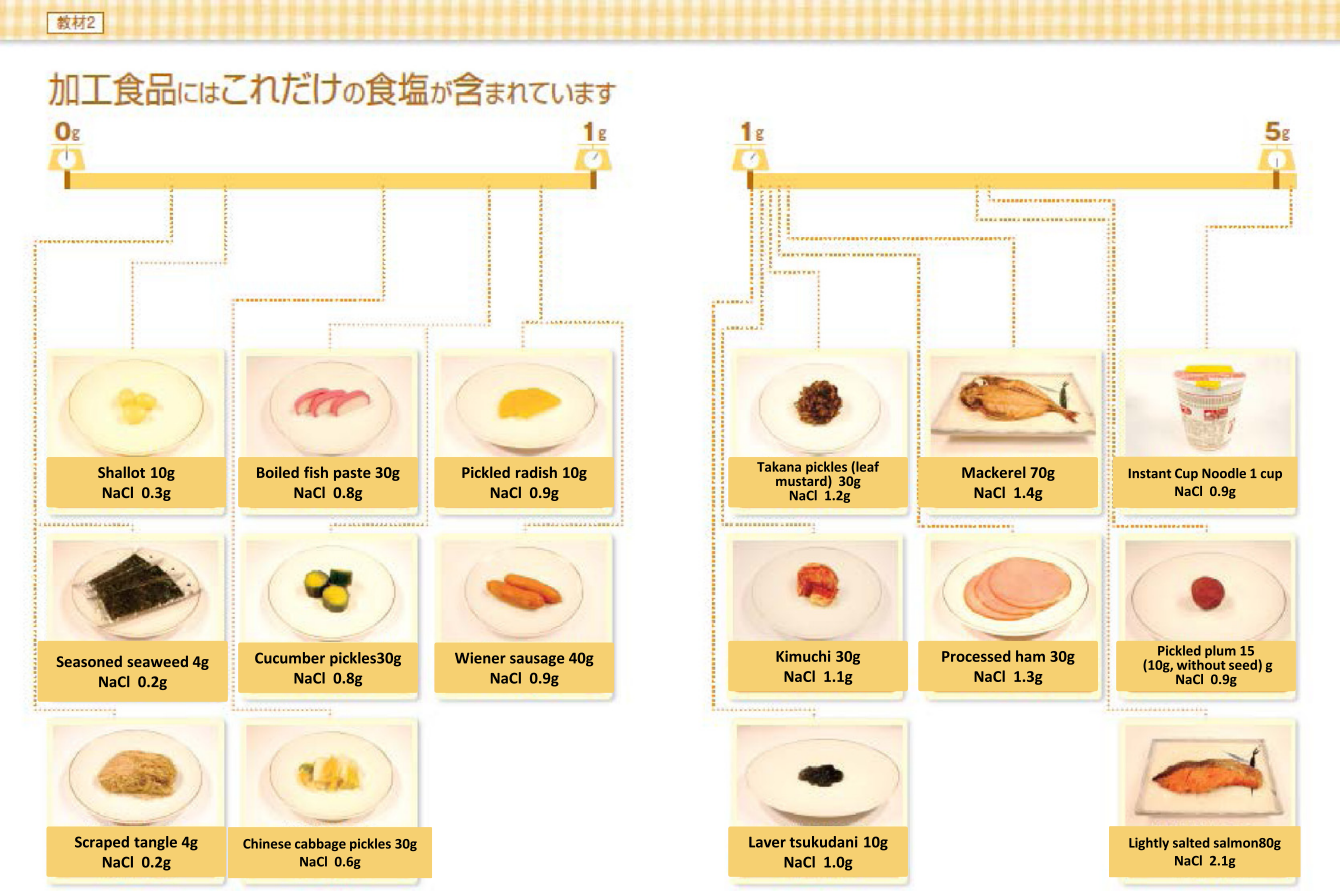
Rough estimate of salt content in the processed foods
  Processed foods are difficult to check. When expressed as salt content as “mg”, salt content should be calculated as: Na (mg) X 2.54/1000 = Salt (g)
- Tips for cooking (Fig. 3)

**Fig. 3:**
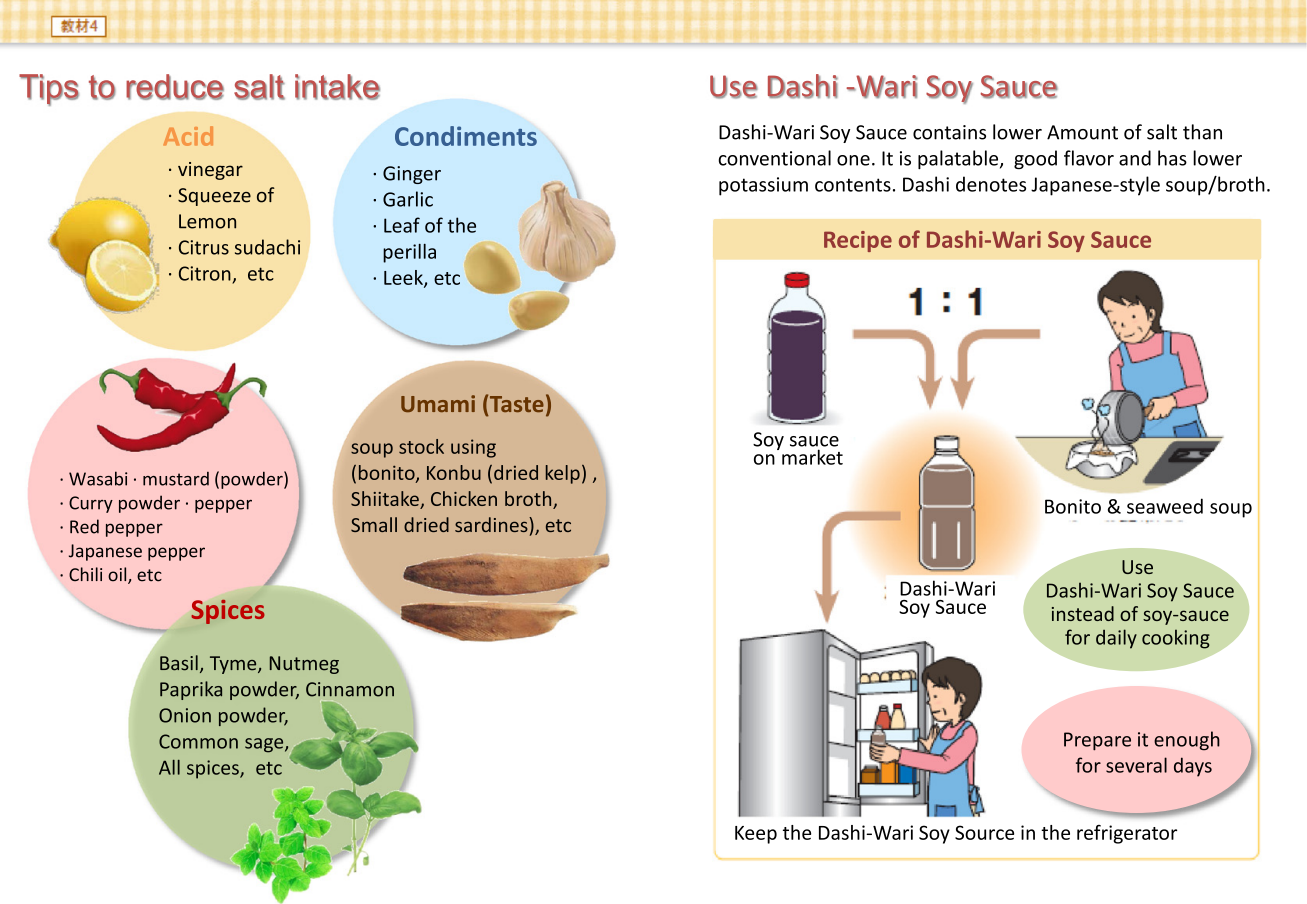
Tips for salt restriction Use other than soy-source
  Salt restricted food is often regarded as “non-palatable”. Adherence to salt restriction could be improved by using other seasoning materials without salt. It may take time to adjust salt restriction.
- Restaurant (Fig. 4)

**Fig. 4:**
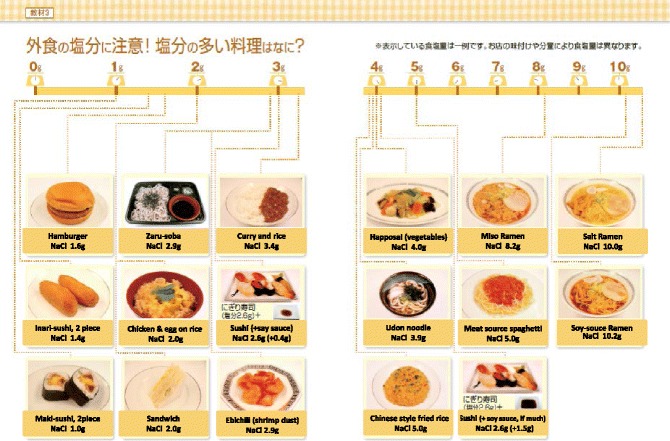
Examples of salty foods at restaurants. (All figures are cited from “Manual for CKD life and dietary guidance manual for physicians and co-medical staffs; edited by the Japanese Society of Nephrology”. Sample Legends: Sample 1. A sample of breakfast. Sample 2. A sample of lunch. Sample 3. A sample of dinner. Sample 4. A sample of balanced diet. (All cited from “Manual for CKD life and dietary guidance manual for physicians and co-medical staffs; edited by the Japanese Society of Nephrology”)
  Generally, cooked-food and diet outside home are salt-rich, although it may differ by restaurant or region. It may be helpful to “recall what and how much have eaten, etc.” Advice to check the nutrient content when buy the cooked foods.

Some tips;

- /Ask low salt cooking at restaurant/Do not drink soup when ordering/
- /Use separate dish for dressing or mayonnaise/Select food easy to estimate salt intake/When eat-out, restrict salt more than usual at home

e) Avoid too much salt restriction
  Too much, less than 3 g/day, salt restriction is dangerous if adequate food intake has not accompanied.

### Protein restriction

1. Rationale
  Ingested protein is finally metabolized to water, carbon dioxide, and other compounds, mainly containing nitrogen. Protein (amino acids) is used to construct body protein and also used for energy production. Water and nitrogen compounds are excreted from kidney. Nitrogenous compounds will retain in the blood as kidney function deteriorates resulting uremic symptoms such as nausea, vomiting, anorexia, and anemia. Electrolyte-imbalance such as hyperkalemia, hyperphosphatemia, and metabolic acidosis may appear in CKD stage 3 patients. To prevent such symptoms, protein restriction is indicated timely in accordance with remaining kidney function to prevent complications and also retard the progression of CKD.
2. Quality of protein
  Human body is constructed by protein such as elastin, collagen, hemoglobin, enzymes, and hormones. Essential amino acid such as leucine, isoleucine, valine, lysine, tryptophan, phenylalanine, threonine, methionine, histidine should be supplied as human cannot synthesize them. Foods with high content of essential amino acid expressed as high amino acid score is regarded as good quality protein food staff. Generally, animal meats are high amino acid score, but not so high in vegetables including soybean products (Table 3).

**Table 3:** Amino-acid score in common food in Japan(1973 FAO/WHO) (Cited with permission from the Japanese Society of Nephrology)

Food	Amino-acid score
Salmon	100
Saury	100
Mackerel	100
Pork, Sirloin	100
Beef	100
Chicken, Round	100
Milk	100
Chicken Egg	100
Polished Rice	65
Soba	65
Sweet Potato	88
Potato	68
Soybean	86
Fermented Soybean (Natto)	84
Okura	57
Green Beans	68
Turnip	45
Asparagus	68
  If protein intake is over 60 g/day, deficiency of essential amino acid is rarely occur. However, in case of protein restriction, insufficient intake of essential amino acid and energy may occur, therefore careful monitoring is recommended to prevent protein-energy wasting (PEW). CKD patients with stage 3b to 5 should be managed with trained dietician and nephrologist.
3. Lipid intake
  Lipid intake should be 20 to 25 % of the total energy intake. N-3 poly-unsaturated fatty acid is recommended for preventing atherosclerosis.
4. Energy intake
  While practicing protein restriction, adequate energy intake should be maintained. Too strict protein restriction may result energy intake deficiency. In particular patients with CKD stage 4 to 5. When energy intake is not sufficient, ingested protein is used for energy, but not for protein construction, resulting muscle and/or wasting. In patients with CKD stage 3b to 5, protein restriction of less than 0.8 g/kg/day should be performed with guidance of special medical team.
5. Practical Guide (Table 4)

**Table 4:** Nutrient content in food common in Japan, expressed grams of food): Food Samples for protein restriction, adjusted to body size (Cited with permission from the Japanese Society of Nephrology)

Height, cm	174	157	152	148
Energy, kcal/day	2000	1800	1600	1400
Protein, g/day	55	45	40	35
Rice,	540	480	390	330
Egg	50	25	25	25
Meat	60	60	50	45
Fish	60	60	50	45
Soybeans	20	20	20	0
Milk Products	120	90	90	90
Vegetables	300	300	300	300
Potato	100	100	100	100
Fruits	120	120	120	120
Sugar/sweets	20	20	20	20
Harusame (Gelatin Noodles)	25	25	25	25
Oils	25	25	25	25
Energy Additive, kcal	100	100	100	100

Three meals a day. Energy Additive; ex) soft-drinks containing carbohydrate 250 ml≑100 kcal
  Patients are instructed to adhere protein restriction by using learning tools to estimate amount of protein, amino acid score, and salt. (Sample 1, 2, 3 and 4)

**Sample 1:**
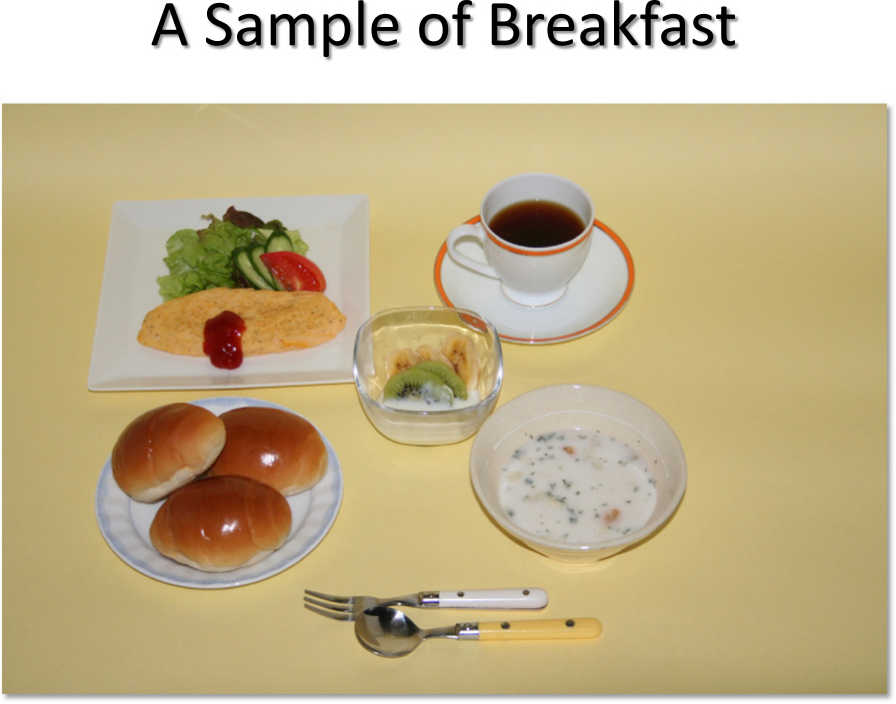
A sample of breakfast

**Sample 2:**
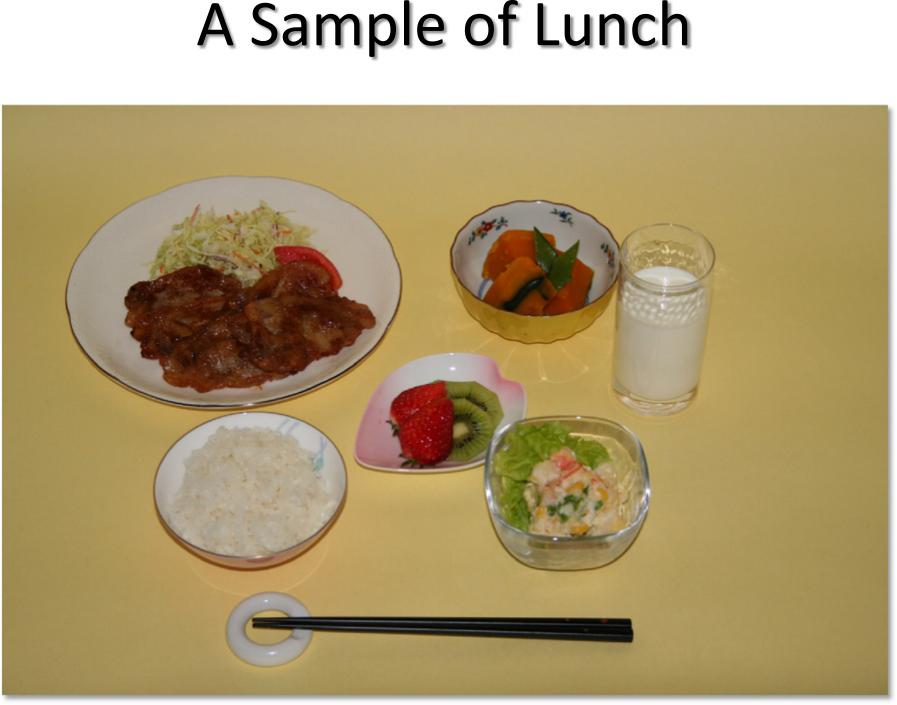
A sample of lunch

**Sample 3:**
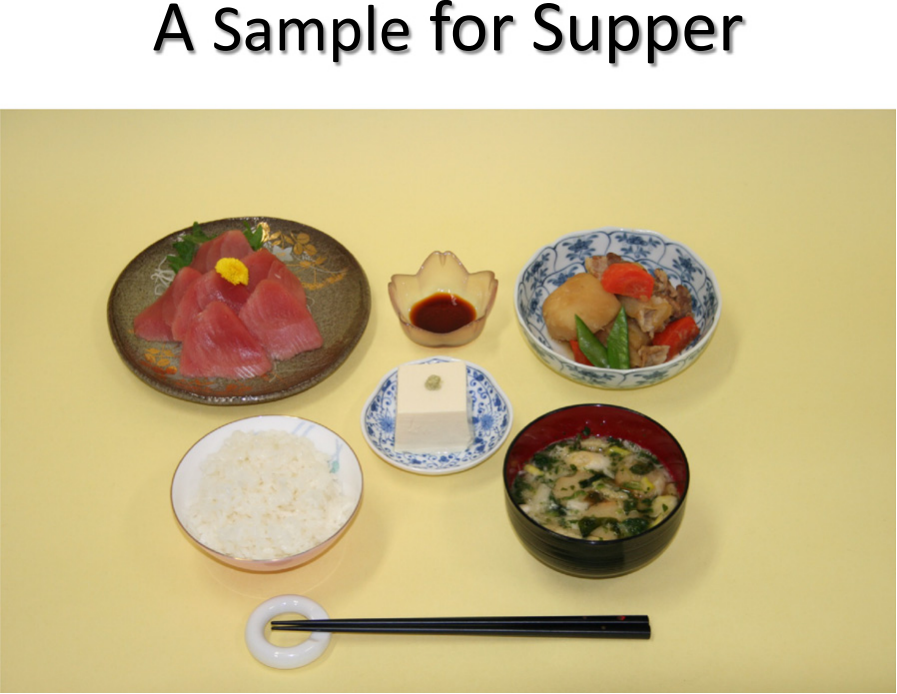
A sample of dinner

**Sample 4:**
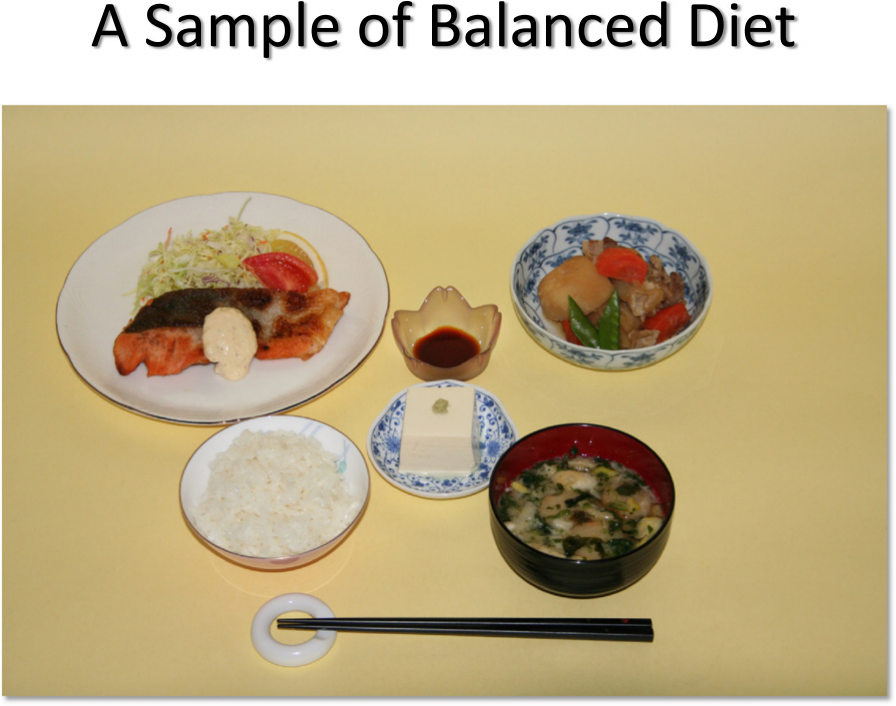
A sample of balanced diet (All cited from “Manual for CKD life and dietary guidance manual for physicians and co-medical staffs; edited by the Japanese Society of Nephrology”)

.

## Summary

We have done a strategic outcome study for chronic kidney disease: Frontier of Renal Outcome Modifications in Japan (FROM-J study) [4]. Management of CKD requires multi-disciplinary involvement. In this study, we prospectively observed the effects of intervention from dietitians and supportive care in CKD patients in addition to usual care recommended by Japanese Society of Nephrology [5]. Through this study, we published practical guidebook with full involvement from dietitian’s society [2, 3].
